# PARP Inhibition Activates STAT3 in Both Tumor and Immune Cells Underlying Therapy Resistance and Immunosuppression In Ovarian Cancer

**DOI:** 10.3389/fonc.2021.724104

**Published:** 2021-12-07

**Authors:** Antons Martincuks, Jieun Song, Adrian Kohut, Chunyan Zhang, Yi-Jia Li, Qianqian Zhao, Edward Mak, Lorna Rodriguez-Rodriguez, Hua Yu, Mihaela Cristea

**Affiliations:** ^1^ Department of Immuno-Oncology, Beckman Research Institute, City of Hope National Medical Center (COH), Duarte, CA, United States; ^2^ Department of Surgery, Division of Gynecologic Oncology, City of Hope National Medical Center (COH), Duarte, CA, United States; ^3^ Department of Medical Oncology & Therapeutics Research, City of Hope National Medical Center (COH), Duarte, CA, United States

**Keywords:** PARP inhibition, STAT3, immunosuppression, ovarian cancer, therapy resistance

## Abstract

Despite the promising activity of poly(ADP-ribose) polymerase (PARP) inhibitors (PARPi) in many cancer types with defects in the DNA damage response the majority of the treated patients acquire PARPi resistance and succumb to their diseases. Consequently, there is an urgent need to identify the mechanisms of PARPi resistance. Here, we show that PARPi treatment promotes STAT3 activation in ovarian cancer cells, tumor-associated immune cells and fibroblasts, resulting in PARPi resistance and immunosuppression. Comparison of ovarian cancer patient-matched tumor biopsies before and after PARPi therapy revealed that STAT3 activity was significantly higher in tumor cells and tumor-associated immune cells and fibroblasts post PARPi treatment. Moreover, one-time PARPi treatment activated STAT3 both in tumor cells as well as diverse immune subsets and fibroblasts. PARPi-treated immune cells exhibited decreased expression of immunostimulatory interferon (IFN)-γ and Granzyme B while increasing immunosuppressive cytokine IL-10. Finally, we demonstrate that the acquisition of PARPi resistance in ovarian cancer cells was accompanied by increased STAT3 activity. Ablating STAT3 inhibited PARPi-resistant ovarian tumor cell growth and/or restored PARPi sensitivity. Therefore, our study has identified a critical mechanism intrinsic to PARPi that promotes resistance to PARPi and induces immunosuppression during PARPi treatment by activating STAT3 in tumor cells and tumor-associated immune cells/fibroblasts.

## Introduction

In recent years, inhibitors of poly(ADP-ribose) polymerase (PARP) have emerged as a promising new therapeutic approach for ovarian cancer treatment, especially for high-grade ovarian carcinoma with mutations in the BRCA1 or BRCA2 tumor suppressor genes (1). PARP inhibition leads to double-stranded DNA breaks during replication, and persistent DNA lesions normally repaired by homologous recombination (HR), causing apoptosis of the BRCA-deficient cancer cells (2, 3). Four PARP inhibitors (PARPi), Olaparib, Rucaparib, Niraparib, and Talazoparib are now FDA-approved for ovarian and breast cancer treatment, and their efficacy depends on BRCA and homologous recombination deficiency (HRD) status, platinum sensitivity, and prior lines of therapy (4). However, despite being one of the most promising new classes of cancer therapeutics, PARPi face enormous hurdles for ovarian cancer treatment. Not all BRCA mutation carriers respond well to PARPi treatment, and among those who achieve an initial response, many eventually become resistant to the drug (5). Therefore, it is necessary to understand the primary and secondary resistance mechanisms to PARPi to improve patient treatment outcomes.

Signal transducer and activator of transcription 3 (STAT3) is a transcription factor that plays a prominent role in promoting tumor cell survival/proliferation and invasion, as well as drug resistance (6, 7). We and others have also demonstrated the importance of STAT3 in suppressing antitumor immune responses in both animal tumor models and clinical studies (8, 9). Persistently activated STAT3 in tumor-associated B cells, CD4+ and CD8+ T cells upregulates expression of immunosuppressive factors while inhibiting immuno-stimulating molecules (10, 11). In ovarian cancer, persistent STAT3 signaling has also been demonstrated to promote tumor survival, invasion, and suppression of antitumor immunity (12, 13), as well as facilitate resistance to several front-line therapeutics, such as cisplatin and paclitaxel (14–16). Recently, it has been demonstrated that both siRNA-mediated and pharmaceutical PARP inhibition leads to an increase in phosphorylation of STAT3 in ovarian cancer cell lines through dePARylation, a critical intrinsic property of PARP blockade, which is responsible for PD-L1 upregulation (17). However, it remains unknown whether PARPi treatment activate STAT3 in ovarian cancer patient tumors to promote therapy resistance, and whether PARPi impact STAT3 activity in tumor-associated immune cells to suppress their ability to mount antitumor immune responses.

In the current study, we investigated whether PARPi treatments in ovarian cancer patients induce STAT3 activation in tumor cells and the tumor immuno-microenvironment and whether PARPi-mediated STAT3 activation underlies PARPi resistance and immunosuppression in ovarian cancer. We provide evidence that post PARPi treatment phosphorylation of STAT3 in germline BRCA ovarian cancer patient biopsies is significantly increased not only in tumor cells but also in tumor-associated diverse immune cells, as well as cancer-associated fibroblasts (CAFs). Furthermore, transient PARPi treatment upregulates STAT3 activation in mouse and human immune cells, leading to enhanced production of immunosuppressive cytokines and reduced expression of immuno-stimulating factors required for T cell-mediated killing of tumor cells. Notably, we show that STAT3 activation is elevated in Olaparib-resistant ovarian cancer cells, and genetic or pharmacological STAT3 inhibition can effectively reduce PARPi-resistant cell proliferation. These results suggest that despite their ability to induce lethality of tumor cells with BRCA mutations, the intrinsic STAT3 activating effects of PARPi can reduce PARPi anti-tumor activity and counteract the efficacies of immunotherapies.

## Material and Methods

### Patients and Tumor Samples

Female ovarian cancer patient specimens were obtained through a City of Hope Institutional Review Board approved protocol. We obtained paired tumor specimens (before and after PARPi therapy) from 6 patients with germline BRCA mutations. Clinical and disease progression data were retrieved from medical records under the same institutionally approved protocol. Samples were de-identified to protect patient confidentiality. Formalin-fixed and paraffin-embedded 4 µm tissue sections were obtained on glass slides for subsequent analysis.

### Antibodies and Reagents

Key reagents used in this work are listed in **Supplementary Table 1**.

### Cell lines and Maintenance of Cultured Cells

A2780, OVCAR8, OVCAR3, SKOV3, and PEO4 human ovarian cancer cells were a generous gift from Dr. Edward Wang, City of Hope Comprehensive Cancer Center, Duarte, CA. PEO1 ovarian cancer cells were purchased from Sigma (#10032308). Mouse embryonic fibroblasts (MEFs) and a non-transformed fibroblast cells (3T3) were a generous gift from Dr. Richard Jove. All cell lines were maintained in Dulbecco’s modified Eagle’s medium (DMEM) supplemented with 2 mM Glutamine, 2 mM Sodium Pyruvate, 10% fetal bovine serum (#FB-12, Omega Scientific), 0.2% MycoZap Plus-CL™ mycoplasma elimination reagent (#VZA-2012, Lonza), and 1% penicillin/streptomycin (#15240-062, Gibco). All cell lines were routinely tested for mycoplasma contamination after every six passages using MycoAlert™ Mycoplasma Detection Kit (#LT07-118, Lonza). Olaparib-resistant A2780 (A2780 Resistant), OVCAR8 (OVCAR8 Resistant) and PEO1 (PEO1 Resistant) cells were generated by continuous incremental drug selection until cells became resistant and were cultivated in 25-32 μM Olaparib.

### *In Vitro* Proliferation Assay

Ovarian cancer cells were plated on a 96-well plate in quadruplicate at 1x10^4^ cells per well overnight. Afterward, cells were treated with the compounds as indicated in figure legends, and cell viability was analyzed using CellTiter-Glo^®^ Luminescent Cell Viability Assay (#G7570, Promega) following the manufacturer’s instructions. Luminescence was recorded using Cytation 5 Cell Imaging Multi-Mode Reader (BioTek).

### Gene Knockdown

For short-term gene silencing, small-interfering RNA (siRNA) targeting STAT3 (#sc-29493, Santa Cruz Biotechnology, Inc) was transfected using RNAiMAX Reagent (#13778030, Thermo Fischer Scientific) according to the manufacturer’s protocol. Unspecific scrambled siRNA (#sc-37007, Santa Cruz Biotechnology, Inc) was used as a control.

### Immunofluorescent Staining and Confocal Microscopy

Formalin-fixed paraffin-embedded ovarian tumor sections were de-paraffinized and dehydrated through xylene and ethanol series, followed by antigen retrieval in high pH Tris-Based Antigen Retrieval Solution (#H-3301, Vector Labs) as per the manufacturer’s protocol and tissue staining using fluorophore-conjugated secondary antibodies was performed as previously described (18). Confocal imaging of immunofluorescence was performed with a Zeiss LSM 880 confocal microscope (Zeiss, Jena, Germany) using 20x and 40x objectives. Staining quantification was performed by ZEN 2.3 lite software and plotted in GraphPad Prism 8 software.

### Real-Time PCR

RNA extraction, reverse transcription and real-time PCR were performed as described previously (11). The relative expression ratio was calculated by the ∆∆Ct method and plotted using GraphPad Prism 8 software.

### Western Blot Analysis

Cultured cells were lysed and analyzed *via* immunoblotting as described elsewhere (19). For signal quantification, the results of three independent experiments were measured using ImageJ (NIH) software and plotted using GraphPad Prism 8 software.

### Isolation of Mouse Immune Cells

Freshly isolated total splenic immune cells were obtained as described before (19). For CD19+ B cell culture, splenic cells were enriched by magnetic beads using negative selection EasySep™ Mouse B Cell Isolation Kit (#19854, StemCell Technologies). Mouse peritoneal macrophages were isolated as described elsewhere (20). All mouse immune cells were cultured in complete RPMI 1640 media containing 10% FBS, 0.2% MycoZap, and 1% penicillin/streptomycin.

### Human Peripheral Blood Mononuclear Cell Preparation and culture

The use of healthy donor blood samples was approved by the City of Hope Institutional Review Board under IRB# 21173 and PBMCs were isolated as described previously (11) cultured in complete RPMI 1640 media containing 10% FBS, 0.2% MycoZap, and 1% penicillin/streptomycin. For tumor-conditioned media (TCM) experiments PBMCs were washed three times with HBSS and incubated with media derived from 48 h culture of indicated ovarian cancer cell lines in full-serum media.

### Cytokine Measurements Using ELISA

The amounts of secreted IL-10 cytokine in human PBMC supernatants and IL-6 cytokine in TCM were measured by a sandwich enzyme-linked immunosorbent assay (ELISA) using human IL-10 (#430604, BioLegend) and human IL-6 (#430504, BioLegend) ELISA kits according to manufacturer’s instructions. Absorbance was read at 450 nm using Cytation 5 Cell Imaging Multi-Mode Reader (BioTek).

### Statistical Analysis

Each experiment was conducted at least three times, and p-values are listed in figure legends. All statistical analyses were performed in Microsoft Excel and GraphPad Prism 8 with data represented as mean ± SD. All statistical comparisons were performed using unpaired two-tailed Student’s t-test, with a p-value of < 0.05 considered statistically significant. Statistical significance was ascribed as ∗p<0.05, ∗∗p<0.01, ∗∗∗p<0.001 and ∗∗∗∗p<0.0001.

## Results

### STAT3 Activity Is Significantly Elevated in Cancer Cells in Ovarian Cancer Patient Tumors After PARPi Treatment

**Figure 1:**
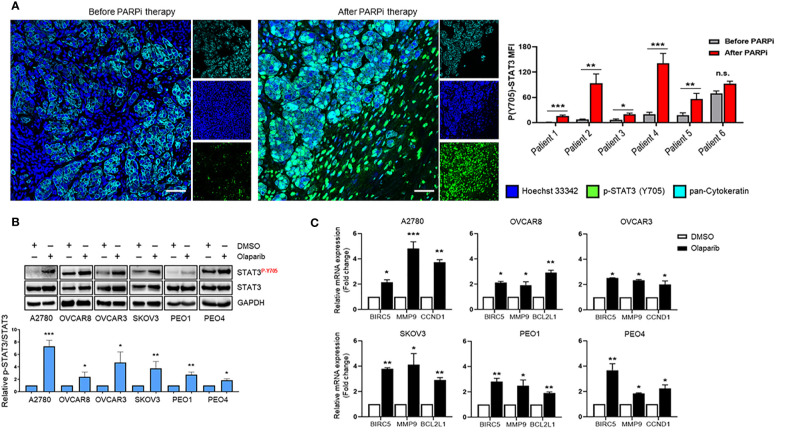
STAT3 phosphorylation is increased in PARPi-treated ovarian cancer patient tumors and cell lines. **(A)** Representative immunofluorescence images (left) from ovarian cancer patients with germline BRCA mutations that were stained for p-STAT3 (green) and pan-Cytokeratin (Cyan), while nuclei were stained with Hoechst (blue). Cytokeratin-stained cell clusters represent malignant tissue. Scale bars = 50 μm. Quantification of p-STAT3 before and after PARPi therapy in BRCA-mutant ovarian patients (right) was performed using ZEN Lite software, normalized to mean nuclear staining, and relative fold change for each patient was plotted using GraphPad Prism 8. Unpaired two-tailed Student t-test was performed against before controls. Shown are means ± SD (n = 9 per sample). *p<0.05, **p<0.01, and ***p<0.001. **(B)** Western blot measuring activated (P-Y705) and total STAT3 levels in A2780, OVCAR8, OVCAR3, SKOV3, PEO1, and PEO4 ovarian cancer cell lines after treatment with 0.1% DMSO or different concentrations of Olaparib (OVCAR3 and PEO1 were treated with 5 µM, A2780 and OVCAR8 with 10 µM, SKOV3 and PEO4 with 20 µ) for 24 h (top). GAPDH immunostaining served as a loading control. Relative band intensities from three independent experiments were quantified using ImageJ software and means are shown ± SD (bottom). Unpaired two-tailed Student t-test of Olaparib treated cells performed against vehicle controls. *p<0.05, **p<0.01, and ***p<0.001. **(C)** Real-time PCR measuring STAT3 target gene *BIRC5, MMP9, CCND1*, and *BCL2L1* mRNA levels in A2780, OVCAR8, OVCAR3, SKOV3, PEO1, and PEO4 ovarian cancer cell lines treated either with DMSO or different concentrations of Olaparib (OVCAR3 and PEO1 were treated with 5 µM, A2780 and OVCAR8 with 10 µM, SKOV3 and PEO4 with 20 µM) for 48 h. Gene expression was normalized to the housekeeper gene *ACTB* and relative expressions against vehicle controls are shown. Unpaired two-tailed Student t-test of Olaparib treated cells performed against DMSO controls. Shown are means ± SD. *p<0.05, **p<0.01, and ***p<0.001, n.s., not significant.

Pharmacologic inhibition of PARP1 with PARPi enhanced the tyrosine 705 (Y705) phosphorylation of STAT3 (p-STAT3) in ovarian cancer cell lines, which was mediated by dePARylation (17). However, it is unknown whether PARPi treatments increase STAT3 activation in ovarian cancer patient tumors. Therefore, we compared p-STAT3 levels in six pairs of patient-matched ovarian carcinoma samples before and after PARPi treatment (three of them received Niraparib, two Rucaparib, and one Olaparib) from patients carrying germline BRCA mutations. Complete treatment overview and timelines for each patient are described in **Supplementary Figure S1**. Immunofluorescence staining was used to assess the levels of p-STAT3 in tumors. The pan-Cytokeratin immunostaining was used to visualize ovarian tumor malignant cell clusters (**Figure 1A** left and **Supplementary Figure S2**). The relative p-STAT3 signal was quantified to assess the changes in STAT3 activation post PARPi treatment (**Figure 1A** right). We detected significantly higher levels of p-STAT3 in ovarian tumors from five out of six patients post PARPi therapy compared to the patient-matched tumor sections prior to PARPi administration. Although no statistically significant difference was observed in the tumor sample of patient #6, the p-STAT3 level was already high before PARPi treatment. These findings suggest that PARPi treatment increases p-STAT3 levels in the tumor cells in BRCA mutated ovarian patients.

### Short-Term Olaparib Treatment Activates STAT3 Signaling in Ovarian Cancer Cell Lines Independent of HR Mutation Status

Although the patient tumors showed significantly elevated p-STAT3 post PARPi treatments, these patients had undergone other treatments before PARPi therapy. To validate a potentially critical role of increasing STAT3 phosphorylation in limiting PARPi therapy efficacy, we tested the effects of Olaparib in multiple human ovarian cancer cell lines. We incubated A2780, OVCAR8, OVCAR3, SKOV3, PEO1, and PEO4 cells with Olaparib and measured STAT3 Y705 phosphorylation changes over vehicle (DMSO) treatment (**Figure 1B**). Our results showed that Olaparib significantly increased STAT3 phosphorylation in all six cell lines, irrespective of BRCA mutation status [BRCA2-mutant PEO1 *vs*. BRCA2-reconstituted PEO4 (21)]. These results demonstrate that Olaparib-mediated increase in STAT3 activation is not a cell-type-specific event and is not dependent on HRD.

Because STAT3 regulates genes that promote cancer cell survival, proliferation, and invasion (8), we next tested mRNA expression changes of previously established STAT3 target genes*, BCL2L1, BIRC5, CCND1*, and *MMP9* after short-term Olaparib exposure (**Figure 1C**). Olaparib increased *BIRC5*, and *MMP9* mRNA levels in all six cell lines, while *CCND1* mRNA levels were upregulated in A2780, OVCAR3 and PEO4 cells, and *BCL2L1* expression levels were significantly elevated in OVCAR8, SKOV3, and PEO1 cell lines. These results collectively show that Olaparib-mediated STAT3 activation leads to upregulation of STAT3-downstream tumorigenic gene expression in ovarian cancer cells with and without HRD.

### STAT3 Signaling Is Upregulated in Olaparib-Resistant Ovarian Cancer Cells

**Figure 2:**
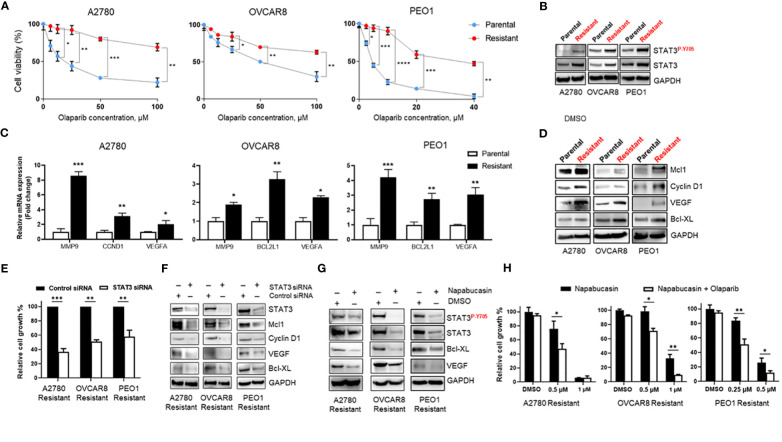
STAT3 activation is elevated in Olaparib-resistant cell lines and genetic or pharmacological STAT3 targeting inhibits Olaparib-resistant tumor cell proliferation **(A)** Drug-response curves of survival after Olaparib treatment of parental and acquired Olaparib-resistant A2780, OVCAR8 and PEO1 cells at 72 h. Shown are representative graphs from three independent experiments as means ± SD (n = 4). Unpaired two-tailed Student t-test of Olaparib-resistant cells performed against parental controls at increasing drug concentrations. Shown are means ± SD. *p<0.05, **p<0.01, ***p<0.001, and ****p<0.0001. **(B)** Western blot showing activated (P-Y705) and total STAT3 levels in parental and Olaparib-resistant A2780, OVCAR8, and PEO1 cells. GAPDH staining served as a loading control. **(C)** Real-time PCR measuring STAT3 target gene *MMP9, CCND1, BCL2L1* and *VEGFA* mRNA levels in parental and Olaparib-resistant A2780, OVCAR8 and PEO1 cells. Gene expression was normalized to the housekeeper gene *ACTB* and relative expressions are shown. Unpaired two-tailed Student t-test of Olaparib-resistant cells performed against parental controls. Shown are means ± SD (n = 3). *p<0.05, **p<0.001, and ***p<0.0001. **(D)** Western blot analyzing STAT3 target gene levels in parental and Olaparib-resistant A2780, OVCAR8, and PEO1 cells. GAPDH staining served as a loading control. **(E)** Cell proliferation analysis of Olaparib-resistant A2780, OVCAR8, and PEO1 cells transfected with either control or STAT3 siRNA. Unpaired two-tailed Student t-test was performed against siRNA controls. Shown are means ± SD (n = 4). **(F)** Western blotting measuring STAT3 target gene levels in Olaparib-resistant A2780, OVCAR8, and PEO1 cells incubated either with either scramble or STAT3 siRNA for 72 h. GAPDH staining served as a loading control. **(G)** Western blotting showing STAT3 target gene levels in Olaparib-resistant A2780, OVCAR8, and PEO1 cells treated either with 0.1% DMSO or Napabucasin (0.5 µM for A2780 Resistant and OVCAR8 Resistant, 0.25 µM for PEO1 Resistant cells) for 72 h. GAPDH staining served as a loading control. **(H)** Cell proliferation assay showing the indicated Napabucasin concentrations kill and sensitize Olaparib-resistant ovarian cancer cells to either 40 µM (for A2780 Resistant and OVCAR8 Resistant) or 10 µM (for PEO1 Resistant) Olaparib. Unpaired two-tailed Student t-test of combinatorial Napabucasin and Olaparib treatment performed against single Napabucasin treatment. Shown are means ± SD (n=3). *p<0.05, **p<0.001.

The mechanisms of PARPi resistance remain poorly understood. Because increased STAT3 signaling has previously been reported in paclitaxel- and cisplatin-resistant ovarian cancer cells (14, 16), we established Olaparib-resistant BRCA-wildtype A2780 (A2780 Resistant), OVCAR8 (OVCAR8 Resistant), and BRCA2-mutated PEO1 (PEO1 Resistant) cell lines as described previously (22) to investigate the potential role of STAT3 in PARPi resistance. A2780 cells share molecular features of BRCA-mutant tumors that correlate with PARPi sensitivity (23), while OVCAR8 cells, despite initial BRCA1 promoter methylation (24), are less sensitive to PARPi due to additional mutations, such as loss of tumor suppressor p53 (23, 25, 26). PEO1 cell line carries a nonsense mutation that generates a truncated BRCA2 form incapable of HR (27), reflecting BRCA2-mutated ovarian cancers. Dose-response curves to Olaparib demonstrated a 7-fold increase in the IC_50_ of BRCA-wildtype Olaparib-resistant A2780 Resistant cells (p<0.001, **Figure 2A** left) and an almost 2-fold increase in OVCAR8 Resistant (p<0.05, **Figure 2A** center) when compared to their parental cells. BRCA2-mutated PEO1 Resistant cells showed a more than 8-fold increase in Olaparib IC_50_ relative to their parental counterparts (p<0.001, **Figure 2A** right). We next tested the changes in phosphorylated and total levels of STAT3 by western blotting of parental and Olaparib-resistant cell lines (**Figure 2B**). Our results revealed that both total and activated STAT3 levels were significantly higher in Olaparib-resistant cells than their parental counterparts.

Importantly, similar to short-term Olaparib treatment (**Figure 1C**), RT-PCR analysis of parental and Olaparib-resistant A2780, OVCAR8, and PEO1 cells showed mRNA upregulation of several essential tumor-promoting genes, such as *MMP9, VEGFA, CCND1*, and *BCL2L1*, which are known STAT3 target genes (8) (**Figure 2C**). Consistent with mRNA data, western blotting of STAT3 target gene protein expression showed that cells with acquired resistance to Olaparib exhibited higher basal levels of Mcl1, Cyclin D1, VEGF, and Bcl-XL proteins compared with parental lines (**Figure 2D**). These data suggest that increased STAT3 signaling is critical for Olaparib resistance in BRCA wild-type and BRCA2-mutated ovarian cancer cells by promoting the expression of genes essential for proliferation and anti-apoptosis.

### Targeting STAT3 Genetically or Pharmacologically Overcomes Ovarian Cancer Cell Olaparib-Resistance

Since both siRNA-mediated and pharmacological STAT3 inhibition has been previously shown to reverse paclitaxel and cisplatin resistance in ovarian cancer cells (14, 15), we tested the reliance of Olaparib-resistant ovarian cancer cells on STAT3 signaling for survival. We first analyzed relative cell growth after transient transfection of either control or STAT3 siRNA for 72h (**Figure 2E**). STAT3 silencing diminished both BRCA-wildtype and BRCA2-mutated Olaparib-resistant cell line proliferation (p<0.001 for A2780 Resistant, p<0.01 for OVCAR8 Resistant and PEO1 Resistant). Moreover, siRNA-mediated STAT3 inhibition reduced the total protein levels of tumor-promoting genes Mcl1, Cyclin D1, VEGF, and Bcl-XL, suggesting that STAT3 is required for proliferation and survival in Olaparib-resistant cells (**Figure 2F**).

To further investigate whether STAT3 targeting could reverse Olaparib resistance in ovarian cancer cells, we incubated Olaparib-resistant ovarian cancer cells for 48 h with the STAT3 inhibitor Napabucasin, which was shown to impair tumor progression and cisplatin resistance (28). Similar to genetic ablation, Napabucasin-mediated STAT3 inhibition led to a significant downregulation of STAT3 target genes, including anti-apoptotic Bcl-XL and pro-angiogenic VEGF at protein level (**Figure 2G**). In parallel, Napabucasin treatment resulted in cell growth inhibition for all three Olaparib-resistant cell lines, and Napabucasin at lower concentrations was able to re-sensitize Olaparib-resistant BRCA-wildtype and BRCA2-mutated cells to Olaparib as demonstrated by cell proliferation assay (**Figure 2H**). These findings indicate that STAT3 activity is required for Olaparib-resistant cell growth and survival, and STAT3 targeting can kill PARPi-resistant ovarian tumor cells and/or restore PARPi sensitivity.

### PARPi Treatment Elevates STAT3 Activity in Patient Ovarian Tumor-Infiltrating Immune Cells and Cancer-Associated Fibroblasts

**Figure 3:**
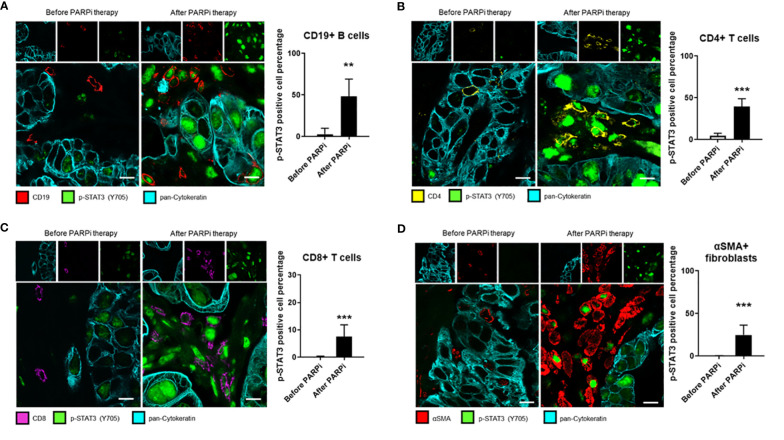
STAT3 activity in ovarian cancer patient tumor-associated immune cells and fibroblasts is elevated post PARPi treatment. Representative immunofluorescence images of tumor sections from six gBRCA ovarian cancer patients before and after PARPi treatment showing p-STAT3 (green), pan-Cytokeratin (cyan), and either CD19+ B cells (red) in **(A)**, CD4+ T cells (yellow) in **(B)**, CD8+ T cells (magenta) in **(C)** or αSMA+ cancer-associated fibroblasts (red) in **(D)**. Cytokeratin-stained cell clusters represent malignant tissue. Scale bars = 10 μm. Histograms (right) showing p-STAT3 expressing cell percentage within indicated populations. Unpaired two-tailed Student t-test performed against the before treatment group. Results represent means ± SD, n = 9 (nine images acquired before and nine images acquired after PARPi treatment from the same patient, six patients total). **p<0.01, ***p<0.001.

Stromal cells, such as immune cells and CAFs, play an important role in tumor progression and resistance to therapies. Although STAT3 signaling in tumor-associated immune cells is known to play a critical role in suppressing antitumor immune responses, few studies have analyzed STAT3 activity in tumor-surrounding lymphocytes in patient tumors before and after specific treatments. To test whether PARPi administration could activate STAT3 in tumor-associated immune cells and thereby limiting PARPi efficacy by promoting immunosuppression directly through immune cells, we performed immunofluorescence staining of specific immune cell subsets from the same six ovarian cancer patients analyzed in **Figure 1A**. Immunofluorescent staining and confocal microscopic analysis of cell surface markers before and after PARPi administration are shown in **Figure 3**. We detected strong p-STAT3 expression in tumor-infiltrating CD19+ B cells, CD4+ T cells, and CD8+ T cells only in post PARPi therapy samples but not in their counterparts before PARPi treatment (**Figures 3A–C**). Moreover, strong p-STAT3 upregulation in immune cells was consistently observed in all six patients (**Supplementary Figures S3–S5**).

Similarly, STAT3 signaling in CAFs has been previously shown to promote stemness, drug resistance, and immunosuppression in several cancer types, including ovarian cancer (29, 30). Therefore, we analyzed the same patient tumor samples for CAF marker αSMA (alpha-smooth muscle actin) expression and p-STAT3. Our results showed that in all six patients, p-STAT3 in αSMA+ CAFs were only detectable in tumor sections post-PARPi treatment, but not in patient-matched biopsies taken before PARPi therapy (**Figure 3D** and **Supplementary Figure S6**). Our findings collectively indicate that in germline BRCA ovarian cancer patients, PARPi treatments significantly elevate STAT3 activity in tumor cells and tumor-associated immune cells, including B cells, CD4+ and CD8+ T cells, and CAFs. Thus, PARPi treatment may limit antitumor immune responses through STAT3 activation in immune cells and CAFs.

### Olaparib Treatment Promotes STAT3 Activation in Immune Cells and Fibroblasts and Subsequent Immunosuppressive Phenotype *In Vitro*

**Figure 4:**
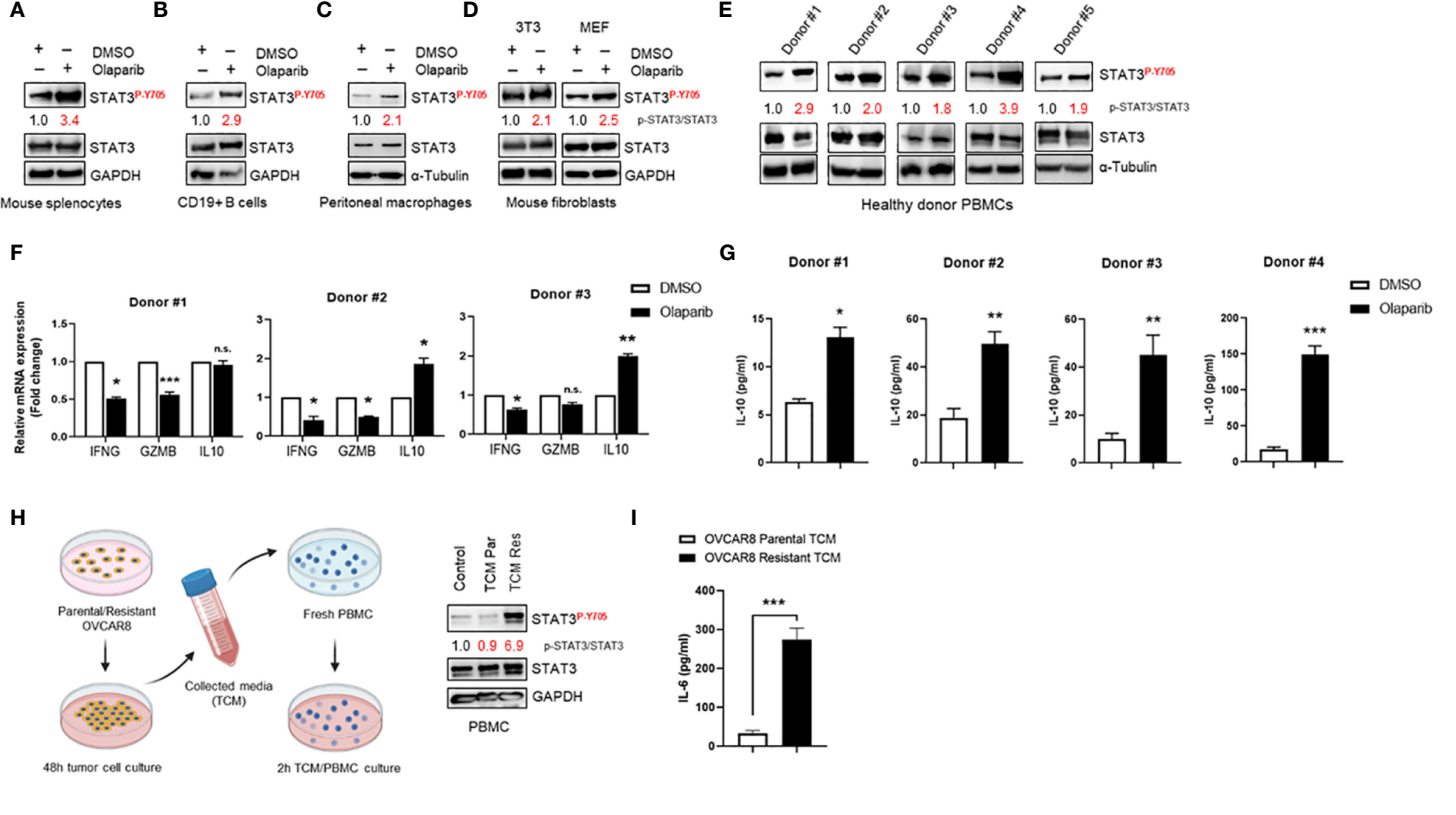
Olaparib activates STAT3 in fibroblasts and immune cells, suppressing immune responses **(A–D)** Western blot showing activated (P-Y705) and total STAT3 levels after Olaparib in freshly isolated splenocytes, CD19+ B cells, and bone marrow-derived macrophages from naive mice, as well as in 3T3 and MEF fibroblasts after treatment with DMSO or 10 µM Olaparib for 24 h. Numbers on the blot show relative activated (P-Y705) vs. total STAT3 ratios as determined by ImageJ. Shown are representative blots of three **(A, D)** or two **(B, C)** independent experiments. GAPDH and α-Tubulin staining served as a loading control. **(E)** Western blotting showing activated (P-Y705) in freshly isolated human PBMCs from five healthy donors treated either with DMSO or 10 μM Olaparib for 48 h. Relative p-STAT3/STAT3 ratios were determined by ImageJ and are shown. α-Tubulin staining served as a loading control. **(F)** Real-time PCR measuring immunostimulatory *IFNG, GZMB* and immune-suppressive *IL10* mRNA levels in human PBMCs from three different donors treated either with DMSO or 10 μM Olaparib for 48 h. Gene expression was normalized to the housekeeper gene *ACTB* and relative expressions against vehicle controls are shown. Unpaired two-tailed Student t-test of Olaparib treatment performed against DMSO controls. Shown are means ± SD (n = 3). *p<0.05, **p<0.01, and ***p<0.001. **(G)** ELISA assessing IL-10 production in the indicated donor PBMC cultures treated either with DMSO or 10 μM Olaparib for 48 h. Unpaired two-tailed Student t-test of Olaparib treatment performed against DMSO controls, shown are means ± SD (n = 3). *p<0.05, **p<0.01, and ***p<0.001 **(H)**. Diagram showing the tumor-conditioned medium (TCM) transfer system (right). Parental or Olaparib-resistant OVCAR8 cells were plated in a 10-cm dish and incubated for 48 h. The supernatant was transferred to a 50-ml centrifuge tube and centrifuged for 5 min. Fresh PBMCs were serum-starved for 24 h, incubated with TCM from parental (TCM Par) or Olaparib-resistant (TCM Res) OVCAR8 cells for 2 h and whole cell lysates were analyzed by Western blotting showing activated (P-Y705) in human PBMCs following TCM incubation (left). For the control, full-serum media was added to PBMCs for 2 h. Activated (P-Y705) vs. total STAT3 ratios were determined by ImageJ. Data are representative of at least three independent experiments. GAPDH served as a loading control. **(I)** ELISA assessing IL-6 production in TCM from parental or Olaparib-resistant OVCAR8 cells. Unpaired two-tailed Student t-test of resistant TCM performed against parental controls, shown are means ± SD (n = 3). ***p<0.001, n.s., not significant.

To confirm and support our findings in ovarian cancer patient tumors, we investigated Olaparib’s effect on STAT3 activation in immune cells and fibroblasts. First, we observed that Olaparib induced STAT3 activation in murine splenocytes (**Figure 4A**). Furthermore, treating CD19+ B cells and peritoneal macrophages from tumor naïve mice with Olaparib also activated STAT3 (**Figures 4B, C**). Additionally, treating mouse 3T3 and MEF fibroblasts with Olaparib increased p-STAT3 (**Figure 4D**).

We next determined whether Olaparib could activate STAT3 in human immune cells. Western blotting to detect p-STAT3 showed that Olaparib treatment of human peripheral blood mononuclear cells (PBMCs) from healthy donors showed increased STAT3 phosphorylation in all six samples tested (**Figure 4E**). These findings further indicate that PARPi-induced STAT3 activation occurs both in tumor cells and tumor microenvironment (TME) components. Furthermore, to explore whether Olaparib-induced STAT3 activation is associated with inhibition of immune responses, we measured total levels of interferon (IFN)-γ, Granzyme B, and IL-10 mRNA in healthy donor PBMC samples upon Olaparib treatment. IFN-γ and Granzyme B are well-known immunostimulatory and antitumor effector molecules produced by cytotoxic T cells (31, 32), both of which are negatively regulated by STAT3. At the same time, IL-10 is an anti-inflammatory cytokine critical for dampening anti-tumor immune responses (33) and upregulated by STAT3. We found that Olaparib significantly lowered IFN-γ (*IFNG*) and Granzyme B (*GZMB*) mRNA levels while increasing immune-suppressive *IL10* expression in all three healthy donors’ PBMCs (**Figure 4F**). Consistent with these results, ELISA analysis of PBMC supernatants revealed a marked increase in IL-10 after Olaparib treatment compared to DMSO-treated control in four different donors (**Figure 4G**). These data indicate that Olaparib treatment promotes immunosuppressive phenotype, as demonstrated by decreased expression of IFN-γ and Granzyme B and increased levels of IL-10 by immune cells. Lastly, we investigated whether increased STAT3 signaling observed in Olaparib-resistant ovarian cancer cell lines can promote paracrine STAT3 activation in tumor microenvironment. We observed that PBMCs incubated with TCM from Olaparib-resistant OVCAR8 cells showed an increase in STAT3 phosphorylation compared to treatment with either control medium or TCM from parental OVCAR8 cells (**Figure 4H**). Given that IL-6 cytokine has been reported as both activator and a downstream target of STAT3 signaling (6), we analyzed the expression of IL-6 as a secreted factor responsible for inducing STAT3 activation within immune cells. Indeed, an IL-6 specific ELISA confirmed that Olaparib-resistant OVCAR8 cells show a significant increase in IL-6 production compared to their parental counterparts (**Figure 4I**).

## Discussion

Although single-agent PARPi have demonstrated significant clinical benefit in ovarian cancer, combination trials of PARPi with other agents have been developed aiming at improving the treatment efficacy. Adding a PARPi to chemotherapy may achieve the efficacy goal, but this approach has been limited by overlapping hematologic toxicity (34). In this context, combined PARP and immune check point inhibition is an attractive strategy. However, despite preclinical studies suggesting that PARPi induce antitumor immune responses, clinical trials have not demonstrated a synergistic effect (35). Therefore, there is an urgent need to understand the molecular mechanisms underlying PARPi resistance and potential immunosuppressive effects that might be intrinsic to PARPi.

For the first time, our studies showed increased STAT3 activation in tumor cells, tumor-associated immune cells, and CAFs in tumor biopsies from ovarian cancer patients that progressed following PARPi treatment. Previously, high levels of activated STAT3 have been found to correlate with advanced-stage ovarian cancer and poor prognosis (36). Our prior work demonstrated increased p-STAT3 positive B cell infiltration in omental tissue (11), and it is associated with poor survival of ovarian cancer patients (37). Furthermore, a recent publication demonstrates that Olaparib-treated macrophages functionally suppress T cell-driven antitumor immune responses (38), and STAT3 activation has been reported to be essential for immune-suppressive macrophage differentiation in advanced epithelial ovarian cancer (39). Lastly, CAFs are known to induce chemoresistance and promote ovarian cancer immune evasion (40, 41), and CAF-intrinsic STAT3 signaling is essential for normal fibroblast transition into CAFs and CAF-mediated tumor progression (29, 30). Therefore, our findings indicate that PARPi treatment-associated p-STAT3 upregulation in ovarian cancer patient tumor-associated immune cells and CAFs may contribute to disease progression by promoting therapy resistance and immunosuppression.

Although our ovarian cancer patient tumor analyses show a statistically significant p-STAT3 upregulation after treatment with PARPi, especially in tumor-infiltrating immune cells and CAFs, these subjects had undergone other treatments prior to PARPi therapy, which could confound the interpretations, and the sample size of paired patient tumor biopsies before and after PARPi therapies is small. It is necessary to assess larger ovarian cancer patient cohorts to confirm the central role of STAT3 in promoting ovarian cancer progression after PARPi treatment. Nevertheless, we show that Olaparib, the most widely used PARPi (42), induced STAT3 activation and expression of several cancer-promoting STAT3 target genes, across several different ovarian cancer cell lines. Short-term Olaparib treatment has also been previously demonstrated to induce cancer cell invasion and enrich PD-L1 and CD44+CD24– stemness marker levels in triple-negative breast cancer (TNBC) cell lines (43). Since CD44+CD24– stem cell-like TNBC phenotype and PD-L1 upregulation require STAT3 signaling (17, 44), these studies support our findings that the STAT3-activating property of PARPi can promote cancer progression and immunosuppression.

Recent publication have reported that Olaparib increased IFN signaling and antitumor immune responses *via* innate immune response cGAS/STING pathway activation and significantly enhanced ICI therapy outcomes in preclinical models (45), which provided sound rationale for combining PARPi with ICI in the clinic. However, in many clinical trials to date, PARPi/ICI combination has not demonstrated significantly beneficial antitumor effects than PARPi or ICI monotherapy (35), suggesting that within patient tumors, additional molecular mechanisms may hinder the synergistic therapeutic response. Importantly, we show that short-term PARPi treatment also decreases IFN-γ and Granzyme B mRNA expression while upregulating IL-10 production by human PBMCs. High IL-10 and low IFN-γ concentrations in ascites from ovarian cancer patients were correlated with poor prognosis and skewed immune cell differentiation towards immune-suppressive and tumor-promoting phenotypes (46, 47). Although there is much to be further explored, our findings indicate that PARP inhibitors possess undesirable properties that limit their antitumor effects, in part by inducing immunosuppression through STAT3 signaling pathway even at the initial stages of PARPi treatments. These findings may also explain the lack of synergistic interaction of PARPi (at least Olaparib) and ICI in cancer patients.

Our studies show that the acquisition of Olaparib resistance in both BRCA-wildtype and BRCA2-mutated ovarian cancer cells is accompanied by increased STAT3 activity and pro-tumorigenic gene expression. Similarly, various receptor tyrosine kinase inhibitors have been previously shown to induce feedback activation of STAT3, which in turn promoted cancer cell survival and targeted therapy resistance (48). Of note, both Y705 phosphorylation and total STAT3 levels were increased in Olaparib-resistant cells, which can be explained by the intrinsic property of activated STAT3 to induce its own expression under certain conditions (49, 50). Likewise, parallel p-STAT3 and total STAT3 upregulation was observed in Paclitaxel and Cisplatin-resistant ovarian cancer cell lines (14, 16), suggesting that elevated and prolonged STAT3 activation increases its own expression in drug-resistant cell lines.

Importantly, STAT3 blockade led to Olaparib-resistant cell growth inhibition, and lower Napabucasin concentrations re-sensitized resistant BRCA-wildtype and BRCA2-mutated ovarian cancer cells to Olaparib *in vitro*, further supporting the notion that STAT3 activity promotes PARPi resistance. In line with our observations, Ruxolitinib, an FDA-approved inhibitor of upstream STAT3 activating JAK1/2 kinases, enhanced sensitivity of myeloproliferative neoplasms to PARPi treatment (51). Similarly, PARPi-resistant TNBC cell lines were re-sensitized to Olaparib with Metformin (43), an anti-diabetic agent known to partially target STAT3, inhibiting TNBC cell proliferation and survival (52).

Although our findings demonstrate an essential role of STAT3 signaling for PARPi resistance acquisition, more studies are required to determine the exact mechanisms by which STAT3 activity facilitates Olaparib-resistant cell survival. One potential STAT3-driven mechanism could be the overexpression of a drug efflux pump MDR1 (ABCB1, P-glycoprotein), which is known to be upregulated upon increased STAT3 activation and expression (53) and has been previously shown to be involved in PARPi resistance *in vitro* and *in vivo* (22, 54). Additionally, Olaparib-resistant cells showed increased pro-angiogenic VEGF, which was reduced by STAT3-siRNA or Napabucasin. Because PARPi combination with VEGF-inhibiting antibody bevacizumab has been shown to significantly improve progression-free survival in an ongoing recurrent ovarian cancer clinical trial (55), STAT3 inhibition may also improve PARPi effects through VEGF downregulation.

Finally, our results further show that STAT3 activation was elevated in the immune cells exposed to PARP inhibitor-resistant tumor cells, which was contributed by an increase in IL-6 expression by Olaparib-resistant OVCAR8 cells relative to their parental counterparts. Our data are in agreement with previous reports showing that p-STAT3-high cell lines from various cancer models secrete higher levels of IL-6 while p-STAT3-low cancer cell lines demonstrate low levels of IL-6 production (56–58). Also, in line with our observations in human PBMCs, tumor-derived factors in conditioned media from tumor cells with elevated STAT3 activity have been shown to promote cancer immunosuppression through dendritic cell maturation and activation inhibition, as well as interfering with leukocyte migration (59–61). Since IL-6 plays an essential role in OvCa microenvironment and is among the most well-known immunosuppressive factors regulated by STAT3 (6, 62), our results show functional consequences of elevated STAT3 signaling in PARP inhibitor-resistant ovarian cancer cells in promoting STAT3 activation within tumor microenvironment. Nevertheless, further studies are needed because there are multiple immunosuppressive factors involved in ovarian cancer immune evasion.

Collectively, our observations suggest that STAT3 inhibition in tumors and tumor-surrounding microenvironment may be a promising strategy to overcome the resistance and improve therapeutic responses of PARPi treatment.

## Data Availability Statement

The original contributions presented in the study are included in the article/**Supplementary Material**. Further inquiries can be directed to the corresponding author.

## Ethics Statement

The studies involving human participants were reviewed and approved by the City of Hope Institutional Review Board. Written informed consent for participation was not required for this study in accordance with the national legislation and the institutional requirements.

## Author Contributions

AM, HY, MC, and LR-R: study conception and design. AM, HY and MC: literature search. MC: provided patient biopsies. AM, JS, AK, CZ, Y-JL, QZ: acquisition of data. AM and HY: data analysis and interpretation. AM: figures. EM: patient data analysis. AM, AK, HY, and MC: drafting the article. AM, JS, AK, CZ, Y-JL, QZ, EM, LR-R, HY, and MC: critical revision of the article and final approval of the version to be published. All authors contributed to the article and approved the submitted version.

## Funding

This work is supported by the Markel-Friedman Accelerator Fund and Women’s Cancers Program at City of Hope, and Grants from Rivkin Center for Ovarian Cancer and the Mary Kay Foundation.

## Conflict of Interest

The authors declare that the research was conducted in the absence of any commercial or financial relationships that could be construed as a potential conflict of interest.

## Publisher’s Note

All claims expressed in this article are solely those of the authors and do not necessarily represent those of their affiliated organizations, or those of the publisher, the editors and the reviewers. Any product that may be evaluated in this article, or claim that may be made by its manufacturer, is not guaranteed or endorsed by the publisher.

## References

[1] Pilie PG Tang C Mills GB Yap TA. State-Of-the-Art Strategies for Targeting the DNA Damage Response in Cancer. Nat Rev Clin Oncol (2019) 16:81–104. doi: 10.1038/s41571-018-0114-z 30356138 PMC8327299

[2] Bryant HE Schultz N Thomas HD Parker KM Flower D Lopez E. Specific Killing of BRCA2-Deficient Tumours With Inhibitors of Poly(ADP-Ribose) Polymerase. Nature (2005) 434:913–7. doi: 10.1038/nature03443 15829966

[3] Farmer H McCabe N Lord CJ Tutt AN Johnson DA Richardson TB. Targeting the DNA Repair Defect in BRCA Mutant Cells as a Therapeutic Strategy. Nature (2005) 434:917–21. doi: 10.1038/nature03445 15829967

[4] Mateo J Lord CJ Serra V Tutt A Balmana J Castroviejo-Bermejo M. A Decade of Clinical Development of PARP Inhibitors in Perspective. Ann Oncol (2019) 30:1437–47. doi: 10.1093/annonc/mdz192 PMC6771225 31218365

[5] Noordermeer SM van Attikum H. PARP Inhibitor Resistance: A Tug-Of-War in BRCA-Mutated Cells. Trends Cell Biol (2019) 29:820–34. doi: 10.1016/j.tcb.2019.07.008 31421928

[6] Yu H Pardoll D Jove R. STATs in Cancer Inflammation and Immunity: A Leading Role for STAT3. Nat Rev Cancer (2009) 9:798–809. doi: 10.1038/nrc2734 19851315 PMC4856025

[7] Zhao C Li H Lin HJ Yang S Lin J Liang G. Feedback Activation of STAT3 as a Cancer Drug-Resistance Mechanism. Trends Pharmacol Sci (2016) 37:47–61. doi: 10.1016/j.tips.2015.10.001 26576830

[8] Yu H Kortylewski M Pardoll D. Crosstalk Between Cancer and Immune Cells: Role of STAT3 in the Tumour Microenvironment. Nat Rev Immunol (2007) 7:41–51. doi: 10.1038/nri1995 17186030

[9] Zou S Tong Q Liu B Huang W Tian Y Fu X. Targeting STAT3 in Cancer Immunotherapy. Mol Cancer (2020) 19:145. doi: 10.1186/s12943-020-01258-7 32972405 PMC7513516

[10] Kortylewski M Kujawski M Wang T Wei S Zhang S Pilon-Thomas S. Inhibiting Stat3 Signaling in the Hematopoietic System Elicits Multicomponent Antitumor Immunity. Nat Med (2005) 11:1314–21. doi: 10.1038/nm1325 16288283

[11] Zhang C Xin H Zhang W Yazaki PJ Zhang Z Le K. CD5 Binds to Interleukin-6 and Induces a Feed-Forward Loop With the Transcription Factor STAT3 in B Cells to Promote Cancer. Immunity (2016) 44:913–23. doi: 10.1016/j.immuni.2016.04.003 PMC4844010 27096320

[12] Saini U Naidu S ElNaggar AC Bid HK Wallbillich JJ Bixel K. Elevated STAT3 Expression in Ovarian Cancer Ascites Promotes Invasion and Metastasis: A Potential Therapeutic Target. Oncogene (2017) 36:168–81. doi: 10.1038/onc.2016.197 PMC5338638 27292260

[13] Wu CJ Sundararajan V Sheu BC Huang RY Wei LH. Activation of STAT3 and STAT5 Signaling in Epithelial Ovarian Cancer Progression: Mechanism and Therapeutic Opportunity. Cancers (Basel) (2019) 12(1):24. doi: 10.3390/cancers12010024 PMC7017004 31861720

[14] Duan Z Foster R Bell DA Mahoney J Wolak K Vaidya A. Signal Transducers and Activators of Transcription 3 Pathway Activation in Drug-Resistant Ovarian Cancer. Clin Cancer Res (2006) 12:5055–63. doi: 10.1158/1078-0432.CCR-06-0861 16951221

[15] Ji T Gong D Han Z Wei X Yan Y Ye F. Abrogation of Constitutive Stat3 Activity Circumvents Cisplatin Resistant Ovarian Cancer. Cancer Lett (2013) 341:231–9. doi: 10.1016/j.canlet.2013.08.022 23962558

[16] Yue P Zhang X Paladino D Sengupta B Ahmad S Holloway RW. Hyperactive EGF Receptor, Jaks and Stat3 Signaling Promote Enhanced Colony-Forming Ability, Motility and Migration of Cisplatin-Resistant Ovarian Cancer Cells. Oncogene (2012) 31:2309–22. doi: 10.1038/onc.2011.409 PMC3245777 21909139

[17] Ding L Chen X Xu X Qian Y Liang G Yao F. PARP1 Suppresses the Transcription of PD-L1 by Poly(ADP-Ribosyl)ating Stat3. Cancer Immunol Res (2019) 7:136–49. doi: 10.1158/2326-6066.CIR-18-0071 30401677

[18] Deng J Liu Y Lee H Herrmann A Zhang W Zhang C. S1PR1-STAT3 Signaling is Crucial for Myeloid Cell Colonization at Future Metastatic Sites. Cancer Cell (2012) 21:642–54. doi: 10.1016/j.ccr.2012.03.039 PMC3360884 22624714

[19] Lee H Deng J Kujawski M Yang C Liu Y Herrmann A. STAT3-Induced S1PR1 Expression is Crucial for Persistent STAT3 Activation in Tumors. Nat Med (2010) 16:1421–8. doi: 10.1038/nm.2250 PMC3088498 21102457

[20] Zhang X Goncalves R Mosser DM. The Isolation and Characterization of Murine Macrophages. Curr Protoc Immunol (2008) 14:14.1. doi: 10.1002/0471142735.im1401s83 PMC2834554 19016445

[21] Sakai W Swisher EM Karlan BY Agarwal MK Higgins J Friedman C. Secondary Mutations as a Mechanism of Cisplatin Resistance in BRCA2-Mutated Cancers. Nature (2008) 451:1116–20. doi: 10.1038/nature06633 PMC2577037 18264087

[22] Vaidyanathan A Sawers L Gannon AL Chakravarty P Scott AL Bray SE. ABCB1 (MDR1) Induction Defines a Common Resistance Mechanism in Paclitaxel- and Olaparib-Resistant Ovarian Cancer Cells. Br J Cancer (2016) 115:431–41. doi: 10.1038/bjc.2016.203 PMC4985349 27415012

[23] McGrail DJ Lin CC Garnett J Liu Q Mo W Dai H. Improved Prediction of PARP Inhibitor Response and Identification of Synergizing Agents Through Use of a Novel Gene Expression Signature Generation Algorithm. NPJ Syst Biol Appl (2017) 3:8. doi: 10.1038/s41540-017-0011-6 28649435 PMC5445594

[24] Stordal B Timms K Farrelly A Gallagher D Busschots S Renaud M. BRCA1/2 Mutation Analysis in 41 Ovarian Cell Lines Reveals Only One Functionally Deleterious BRCA1 Mutation. Mol Oncol (2013) 7:567–79. doi: 10.1016/j.molonc.2012.12.007 PMC4106023 23415752

[25] Arizti P Fang L Park I Yin Y Solomon E Ouchi T. Tumor Suppressor P53 is Required to Modulate BRCA1 Expression. Mol Cell Biol (2000) 20:7450–9. doi: 10.1128/MCB.20.20.7450-7459.2000 PMC86298 11003642

[26] Leroy B Girard L Hollestelle A Minna JD Gazdar AF Soussi T. Analysis of TP53 Mutation Status in Human Cancer Cell Lines: A Reassessment. Hum Mutat (2014) 35:756–65. doi: 10.1002/humu.22556 PMC4451114 24700732

[27] Sakai W Swisher EM Jacquemont C Chandramohan KV Couch FJ Langdon SP. Functional Restoration of BRCA2 Protein by Secondary BRCA2 Mutations in BRCA2-Mutated Ovarian Carcinoma. Cancer Res (2009) 69:6381–6. doi: 10.1158/0008-5472.CAN-09-1178 PMC2754824 19654294

[28] MacDonagh L Gray SG Breen E Cuffe S Finn SP O'Byrne KJ. BBI608 Inhibits Cancer Stemness and Reverses Cisplatin Resistance in NSCLC. Cancer Lett (2018) 428:117–26. doi: 10.1016/j.canlet.2018.04.008 29653268

[29] Wang Y Jing Y Ding L Zhang X Song Y Chen S. Epiregulin Reprograms Cancer-Associated Fibroblasts and Facilitates Oral Squamous Cell Carcinoma Invasion *via* JAK2-STAT3 Pathway. J Exp Clin Cancer Res (2019) 38:274. doi: 10.1186/s13046-019-1277-x 31234944 PMC6591968

[30] Yang X Lin Y Shi Y Li B Liu W Yin W. FAP Promotes Immunosuppression by Cancer-Associated Fibroblasts in the Tumor Microenvironment *via* STAT3-CCL2 Signaling. Cancer Res (2016) 76:4124–35. doi: 10.1158/0008-5472.CAN-15-2973 27216177

[31] Badovinac VP Tvinnereim AR Harty JT. Regulation of Antigen-Specific CD8+ T Cell Homeostasis by Perforin and Interferon-Gamma. Science (2000) 290:1354–8. doi: 10.1126/science.290.5495.1354 11082062

[32] Trapani JA Sutton VR Granzyme B. Pro-Apoptotic, Antiviral and Antitumor Functions. Curr Opin Immunol (2003) 15:533–43. doi: 10.1016/S0952-7915(03)00107-9 14499262

[33] Dennis KL Blatner NR Gounari F Khazaie K. Current Status of Interleukin-10 and Regulatory T-Cells in Cancer. Curr Opin Oncol (2013) 25:637–45. doi: 10.1097/CCO.0000000000000006 PMC4322764 24076584

[34] Kunos C Deng W Dawson D Lea JS Zanotti KM Gray HJ. A Phase I-II Evaluation of Veliparib (NSC #737664), Topotecan, and Filgrastim or Pegfilgrastim in the Treatment of Persistent or Recurrent Carcinoma of the Uterine Cervix: An NRG Oncology/Gynecologic Oncology Group Study. Int J Gynecol Cancer (2015) 25:484–92. doi: 10.1097/IGC.0000000000000380 PMC4336206 25594147

[35] Takahashi N Surolia I Thomas A. Targeting DNA Repair to Drive Immune Responses: It's Time to Reconsider the Strategy for Clinical Translation. Clin Cancer Res (2020) 26:2452–6. doi: 10.1158/1078-0432.CCR-19-3841 PMC8283908 32066627

[36] Silver DL Naora H Liu J Cheng W Montell DJ. Activated Signal Transducer and Activator of Transcription (STAT) 3: Localization in Focal Adhesions and Function in Ovarian Cancer Cell Motility. Cancer Res (2004) 64:3550–8. doi: 10.1158/0008-5472.CAN-03-3959 15150111

[37] Yang C Lee H Jove V Deng J Zhang W Liu X. Prognostic Significance of B-Cells and Pstat3 in Patients With Ovarian Cancer. PloS One (2013) 8:e54029. doi: 10.1371/journal.pone.0054029 23326565 PMC3542323

[38] Mehta AK Cheney EM Hartl CA Pantelidou C Oliwa M Castrillon JA. Targeting Immunosuppressive Macrophages Overcomes PARP Inhibitor Resistance in BRCA1-Associated Triple-Negative Breast Cancer. Nat Cancer (2021) 2:66–82. doi: 10.1038/s43018-020-00148-7 33738458 PMC7963404

[39] Takaishi K Komohara Y Tashiro H Ohtake H Nakagawa T Katabuchi H. Involvement of M2-Polarized Macrophages in the Ascites From Advanced Epithelial Ovarian Carcinoma in Tumor Progression *via* Stat3 Activation. Cancer Sci (2010) 101:2128–36. doi: 10.1111/j.1349-7006.2010.01652.x PMC11159803 20860602

[40] Wang W Kryczek I Dostál L Lin H Tan L Zhao L. Effector T Cells Abrogate Stroma-Mediated Chemoresistance in Ovarian Cancer. Cell (2016) 165:1092–105. doi: 10.1016/j.cell.2016.04.009 PMC4874853 27133165

[41] Dasari S Fang Y Mitra AK. Cancer Associated Fibroblasts: Naughty Neighbors That Drive Ovarian Cancer Progression. Cancers (Basel) (2018) 10(11):406. doi: 10.3390/cancers10110406 PMC6265896 30380628

[42] Heo YA Dhillon S. Olaparib Tablet: A Review in Ovarian Cancer Maintenance Therapy. Target Oncol (2018) 13:801–8. doi: 10.1007/s11523-018-0606-x 30456461

[43] Han Y Li CW Hsu JM Hsu JL Chan LC Tan X. Metformin Reverses PARP Inhibitors-Induced Epithelial-Mesenchymal Transition and PD-L1 Upregulation in Triple-Negative Breast Cancer. Am J Cancer Res (2019) 9:800–15. PMC6511636 31106005

[44] Marotta LL Almendro V Marusyk A Shipitsin M Schemme J Walker SR. The JAK2/STAT3 Signaling Pathway is Required for Growth of CD44⁺CD24⁻ Stem Cell-Like Breast Cancer Cells in Human Tumors. J Clin Invest (2011) 121:2723–35. doi: 10.1172/JCI44745 PMC3223826 21633165

[45] Pantelidou C Sonzogni O De Oliveria Taveira M Mehta AK Kothari A Wang D. PARP Inhibitor Efficacy Depends on CD8(+) T-Cell Recruitment *via* Intratumoral STING Pathway Activation in BRCA-Deficient Models of Triple-Negative Breast Cancer. Cancer Discov (2019) 9:722–37. doi: 10.1158/2159-8290.CD-18-1218 PMC6548644 31015319

[46] Jeannin P Duluc D Delneste Y. IL-6 and Leukemia-Inhibitory Factor are Involved in the Generation of Tumor-Associated Macrophage: Regulation by IFN-γ. Immunotherapy (2011) 3:23–6. doi: 10.2217/imt.11.30 21524164

[47] Wu L Deng Z Peng Y Han L Liu J Wang L. Ascites-Derived IL-6 and IL-10 Synergistically Expand CD14(+)HLA-DR(-/Low) Myeloid-Derived Suppressor Cells in Ovarian Cancer Patients. Oncotarget (2017) 8:76843–56. doi: 10.18632/oncotarget.20164 PMC5652747 29100353

[48] Lee HJ Zhuang G Cao Y Du P Kim HJ Settleman J. Drug Resistance *via* Feedback Activation of Stat3 in Oncogene-Addicted Cancer Cells. Cancer Cell (2014) 26:207–21. doi: 10.1016/j.ccr.2014.05.019 25065853

[49] Ichiba M Nakajima K Yamanaka Y Kiuchi N Hirano T. Autoregulation of the Stat3 Gene Through Cooperation With a cAMP-Responsive Element-Binding Protein. J Biol Chem (1998) 273:6132–8. doi: 10.1074/jbc.273.11.6132 9497331

[50] Narimatsu M Maeda H Itoh S Atsumi T Ohtani T Nishida K. Tissue-Specific Autoregulation of the Stat3 Gene and Its Role in Interleukin-6-Induced Survival Signals in T Cells. Mol Cell Biol (2001) 21:6615–25. doi: 10.1128/MCB.21.19.6615-6625.2001 PMC99807 11533249

[51] Nieborowska-Skorska M Maifrede S Dasgupta Y Sullivan K Flis S Le BV. Ruxolitinib-Induced Defects in DNA Repair Cause Sensitivity to PARP Inhibitors in Myeloproliferative Neoplasms. Blood (2017) 130:2848–59. doi: 10.1182/blood-2017-05-784942 PMC5746670 29042365

[52] Deng XS Wang S Deng A Liu B Edgerton SM Lind SE. Metformin Targets Stat3 to Inhibit Cell Growth and Induce Apoptosis in Triple-Negative Breast Cancers. Cell Cycle (2012) 11:367–76. doi: 10.4161/cc.11.2.18813 22189713

[53] Wang Z Wang C Zuo D Zhang T Yin F Zhou Z. Attenuation of STAT3 Phosphorylation Promotes Apoptosis and Chemosensitivity in Human Osteosarcoma Induced by Raddeanin a. Int J Biol Sci (2019) 15:668–79. doi: 10.7150/ijbs.30168 PMC6367581 30745853

[54] Kim H Xu H George E Hallberg D Kumar S Jagannathan V. Combining PARP With ATR Inhibition Overcomes PARP Inhibitor and Platinum Resistance in Ovarian Cancer Models. Nat Commun (2020) 11:3726. doi: 10.1038/s41467-020-17127-2 32709856 PMC7381609

[55] Mirza MR Åvall Lundqvist E Birrer MJ dePont Christensen R Nyvang GB Malander S. Niraparib Plus Bevacizumab Versus Niraparib Alone for Platinum-Sensitive Recurrent Ovarian Cancer (NSGO-AVANOVA2/ENGOT-Ov24): A Randomised, Phase 2, Superiority Trial. Lancet Oncol (2019) 20:1409–19. doi: 10.1016/S1470-2045(19)30515-7 31474354

[56] Lieblein JC Ball S Hutzen B Sasser AK Lin HJ Huang TH. STAT3 can be Activated Through Paracrine Signaling in Breast Epithelial Cells. BMC Cancer (2008) 8:302. doi: 10.1186/1471-2407-8-302 18939993 PMC2582243

[57] Griesinger AM Josephson RJ Donson AM Mulcahy Levy JM Amani V Birks DK. Interleukin-6/STAT3 Pathway Signaling Drives an Inflammatory Phenotype in Group A Ependymoma. Cancer Immunol Res (2015) 3:1165–74. doi: 10.1158/2326-6066.CIR-15-0061 PMC4596749 25968456

[58] Morgan EL Macdonald A. Autocrine STAT3 Activation in HPV Positive Cervical Cancer Through a Virus-Driven Rac1-Nfκb-IL-6 Signalling Axis. PloS Pathog (2019) 15:e1007835. doi: 10.1371/journal.ppat.1007835 31226168 PMC6608985

[59] Nefedova Y Huang M Kusmartsev S Bhattacharya R Cheng P Salup R. Hyperactivation of STAT3 is Involved in Abnormal Differentiation of Dendritic Cells in Cancer. J Immunol (2004) 172:464–74. doi: 10.4049/jimmunol.172.1.464 14688356

[60] Bharadwaj U Li M Zhang R Chen C Yao Q. Elevated Interleukin-6 and G-CSF in Human Pancreatic Cancer Cell Conditioned Medium Suppress Dendritic Cell Differentiation and Activation. Cancer Res (2007) 67:5479–88. doi: 10.1158/0008-5472.CAN-06-3963 17545630

[61] Albesiano E Davis M See AP Han JE Lim M Pardoll DM. Immunologic Consequences of Signal Transducers and Activators of Transcription 3 Activation in Human Squamous Cell Carcinoma. Cancer Res (2010) 70:6467–76. doi: 10.1158/0008-5472.CAN-09-4058 PMC2922407 20682796

[62] Coward J Kulbe H Chakravarty P Leader D Vassileva V Leinster DA. Interleukin-6 as a Therapeutic Target in Human Ovarian Cancer. Clin Cancer Res (2011) 17:6083–96. doi: 10.1158/1078-0432.CCR-11-0945 PMC3182554 21795409

