# Supplementary material for: PARP Inhibition Activates STAT3 in Both Tumor and Immune Cells Underlying Therapy Resistance and Immunosuppression In Ovarian Cancer
Source: Front Oncol. 2021 Dec 7;11:724104. doi: 10.3389/fonc.2021.724104 (PMC8693573; doi:10.3389/fonc.2021.724104)
Supplement: Supplementary file 1 [file DataSheet_1.pdf]

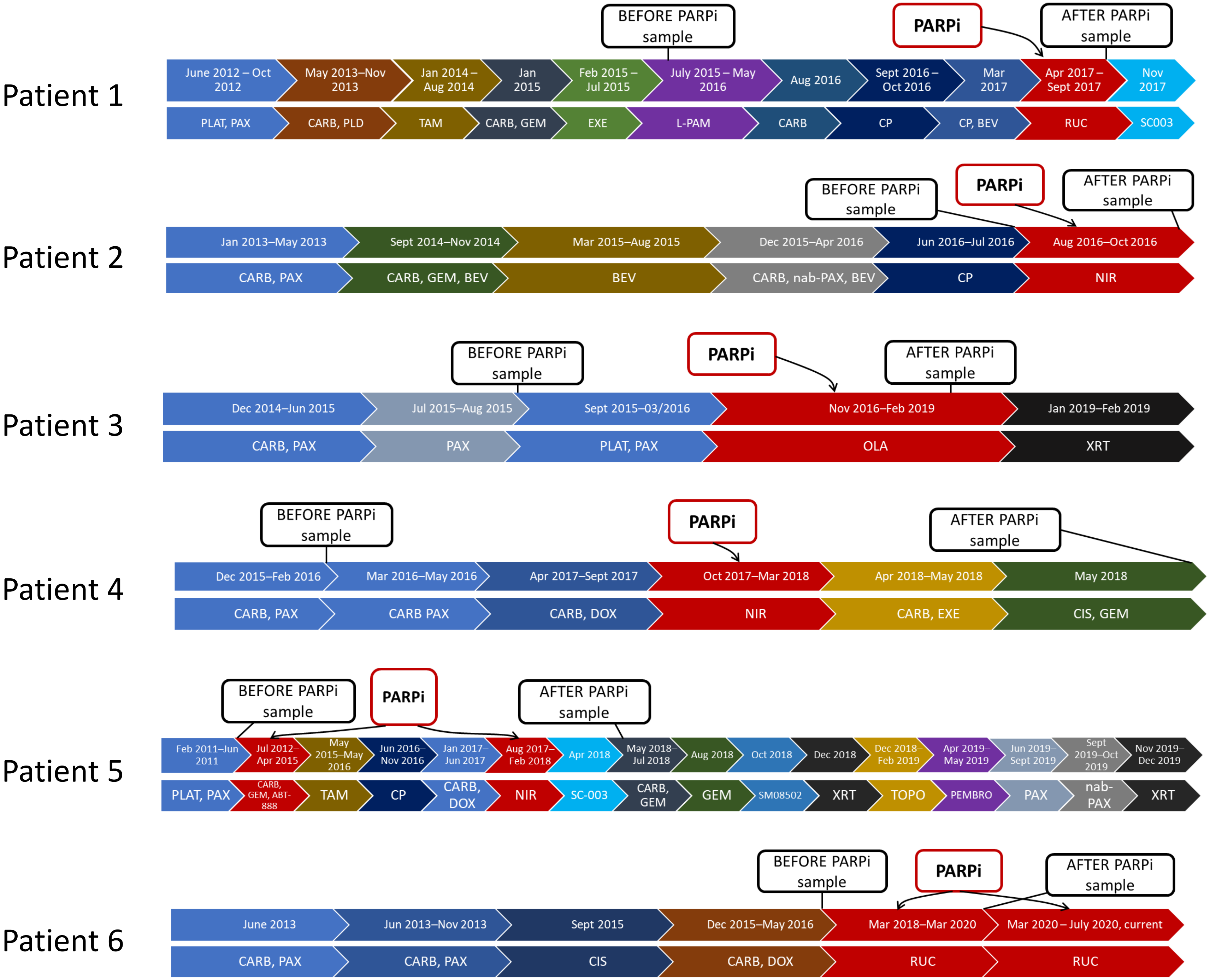

Supplemental Figure S1. Martincuks, A et al.

Supplemental Figure S1. Ovarian cancer patient treatment timelines. Related to Figures 1A and 4. The treatment history of six germline BRCA ovarian cancer patients used in this study. Indicated are time points when "Before PARPi" and "After PARPi" samples were taken, as well as the exact period of PARPi treatment course. Abbreviations: CIS, cisplatin; PLAT, platinum; PAX, paclitaxel; CARB, carboplatin; PLD, pegylated liposomal doxorubicin; TAM, tamoxifen; GEM, gemcitabine; EXE, exemestane; L-PAM, melphalan; CP, cyclophosphamide; BEV, bevacizumab; RUC, rucaparib; SC003, SC-003 antibody-drug conjugate; ABT-888, veliparib; DOX, doxorubicin; NIR, niraparib; SM08502, SM08502 CLK inhibitor; XRT, radiation, TOPO, topotecan, PEMBRO, pembrolizumab; nab-PAX, nab-paclitaxel; OLA, olaparib.

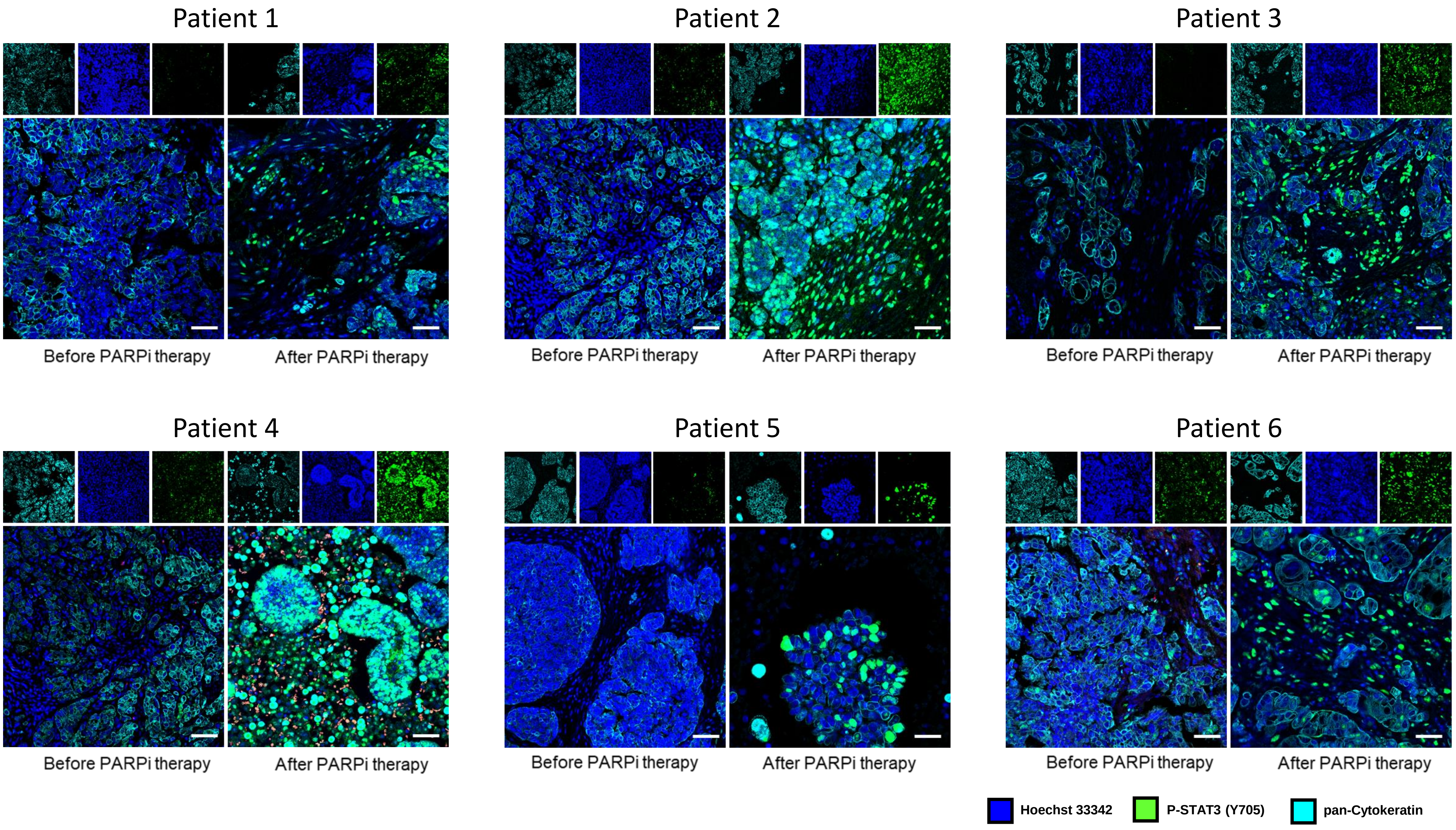

Supplemental Figure S2. Martincuks, A et al.

Supplemental Figure S2. An increase in p-STAT3 is found in gBRCA ovarian cancer patient tumor samples post PARPi treatments. Related to Figure 1. Representative immunofluorescence images for each of the six ovarian cancer patients with germline BRCA mutations that were stained for p-STAT3 (green), pan-Cytokeratin (cyan), and Hoechst nuclear staining (blue). Scale bars = 50 μm

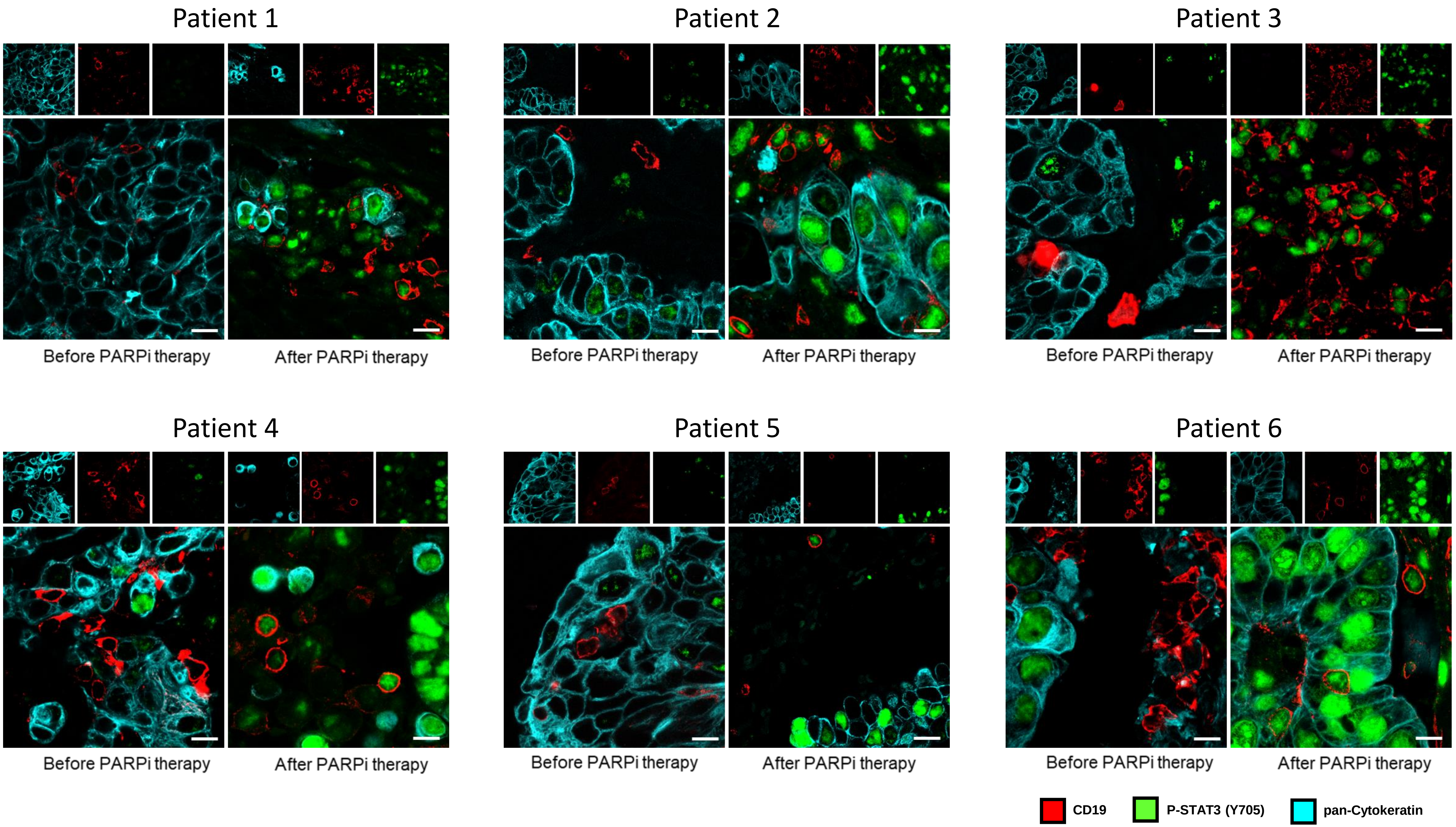

Supp. Fig. 3

Supplemental Figure S3. Martincuks, A et al.

Supplemental Figure S3. PARPi treatment correlates with p-STAT3 increase in CD19+ B cells in gBRCA ovarian cancer patient tumor samples. Related to Figure 4A. Representative immunofluorescence images for each of the six ovarian cancer patients that were stained for p-STAT3 (green), pan-Cytokeratin (cyan), and CD19 (red). Scale bars = 10 μm.

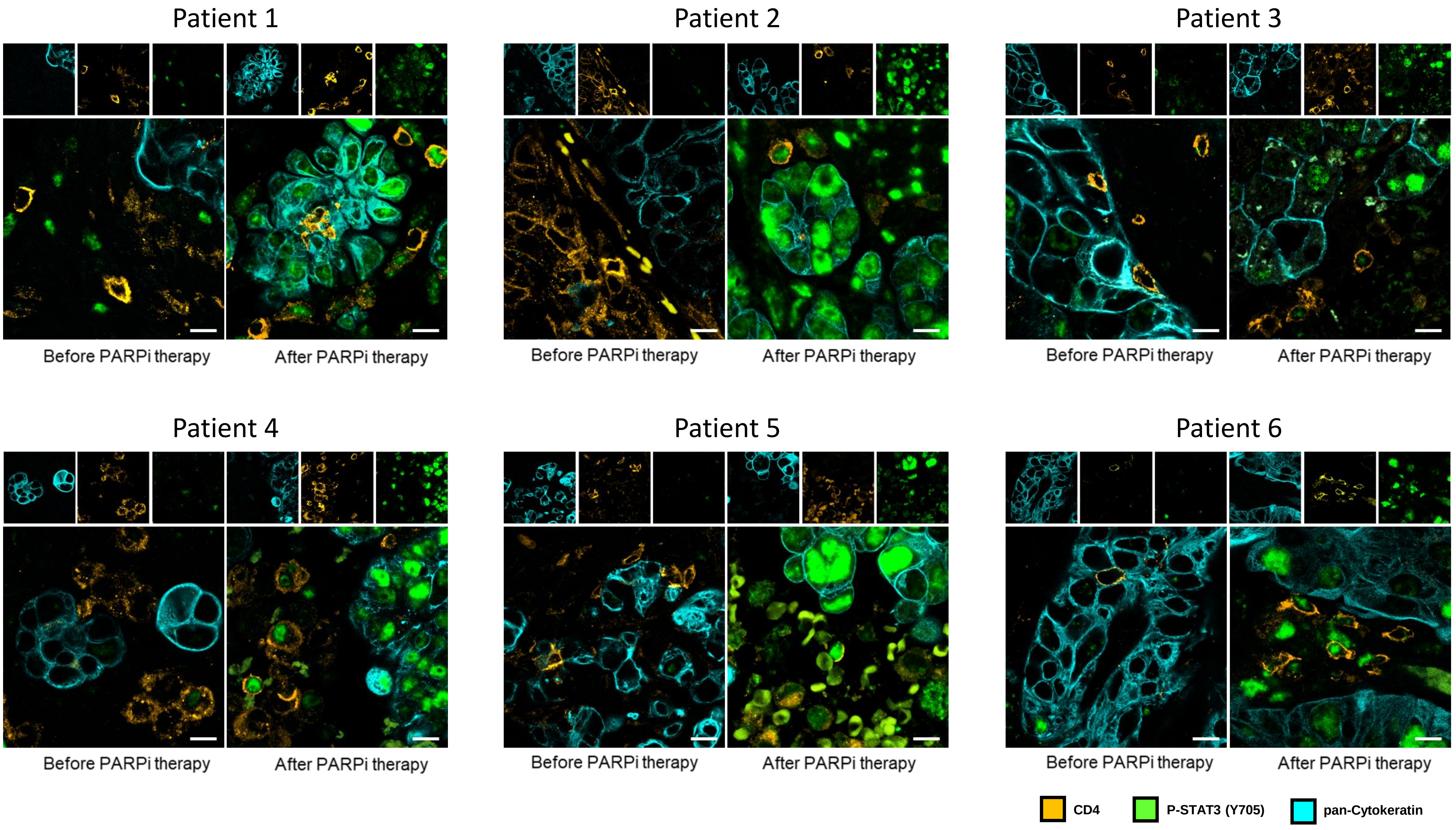

Supp. Fig. 4

Supplemental Figure S4. Martincuks, A et al.

Supplemental Figure S4. PARPi treatment correlates with p-STAT3 increase in CD4+ T cells in gBRCA ovarian cancer patient tumors. Related to Figure 4B. Representative immunofluorescence images for each of the six ovarian cancer patients that were stained for p-STAT3 (green), pan-Cytokeratin (cyan), and CD4 (yellow). Scale bars = 10 μm.

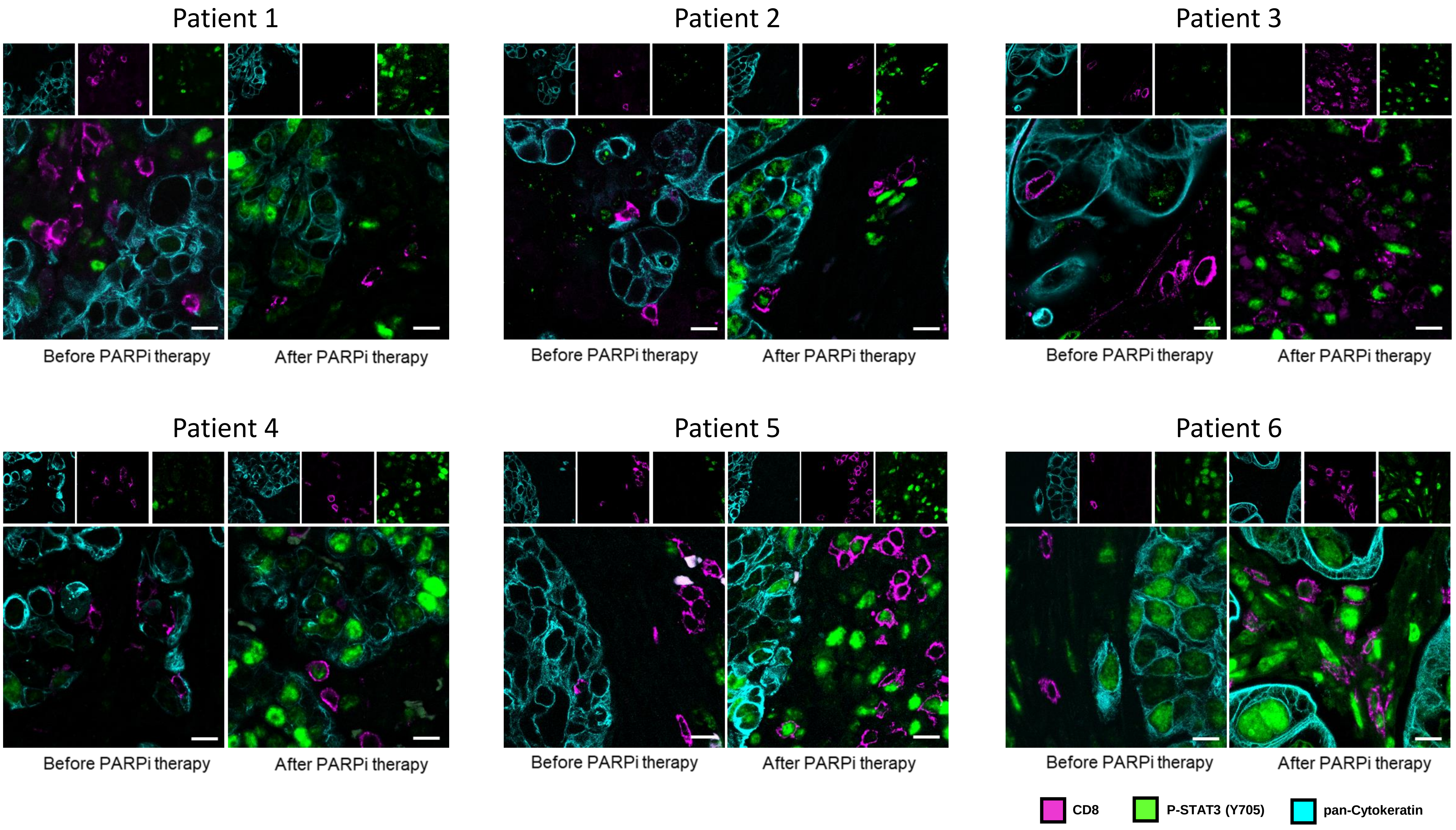

Supp. Fig. 5

Supplemental Figure S5. Martincuks, A et al.

Supplemental Figure S5. p-STAT3 is increased in post-PARPi treatment patient tumor CD8+ T cells. Related to Figure 4C. Representative immunofluorescence images for each of the six pairs of ovarian cancer patient tumors that were stained for p-STAT3 (green), pan-Cytokeratin (cyan), and CD8 (magenta). Scale bars = 10 µm.

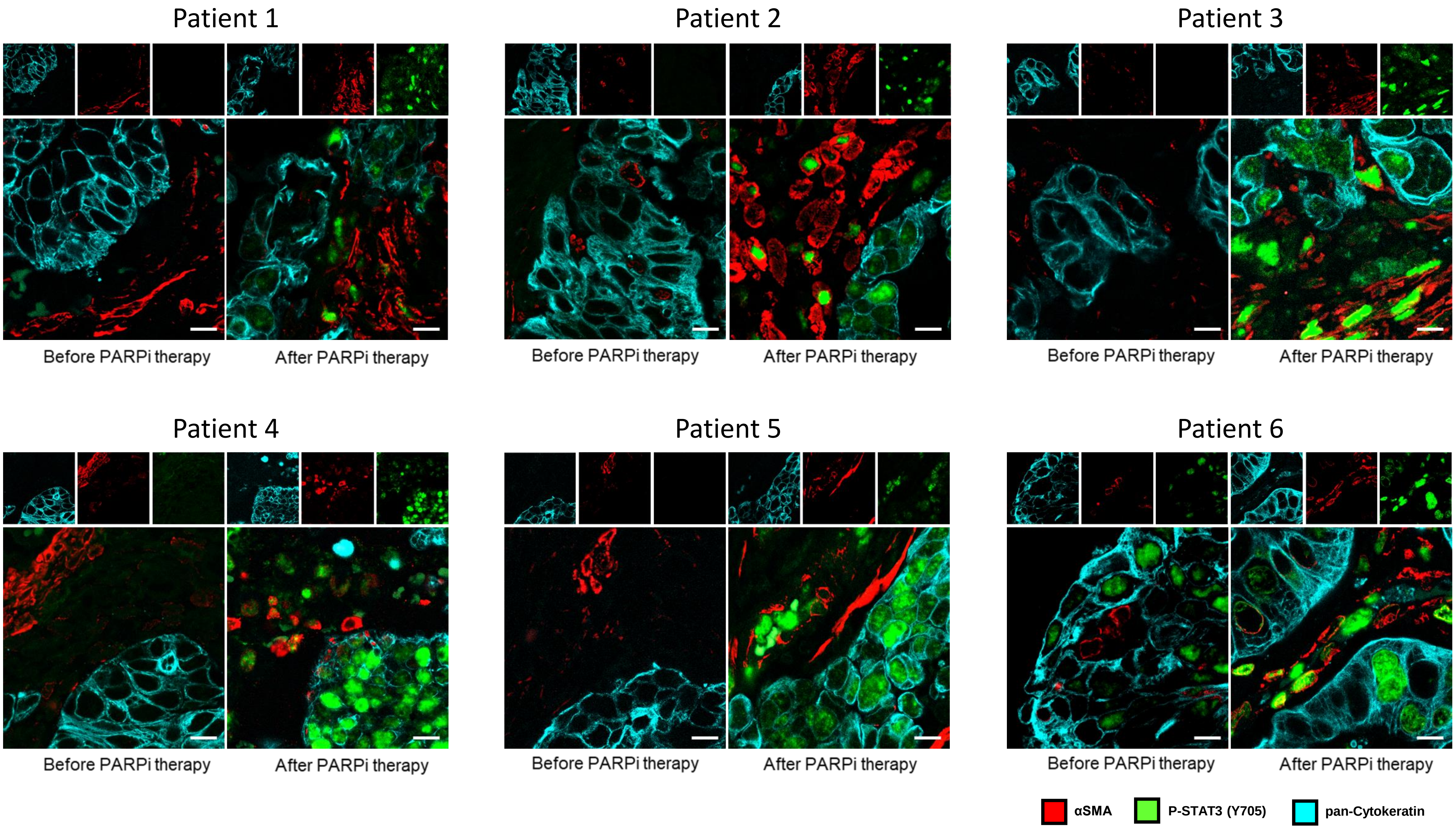

Supp. Fig. 6

Supplemental Figure S6. Martincuks, A et al.

Supplemental Figure S6. PARPi treatment correlates with p-STAT3 increase in α-SMA+ fibroblasts in gBRCA ovarian cancer patient tumor samples. Related to Figure 4D. Representative immunofluorescence images for each of the six ovarian cancer patients that were stained for p-STAT3 (green), pan-Cytokeratin (cyan), and α-SMA (red). Scale bars =10 μm.

Supplementary Tables

Supplementary Table 1. Key resources table.

Reagent	Manufacturer	Cat. Number
Primary antibodies		
anti-pY705-STAT3, rabbit monoclonal (for Western blot)	Cell Signalling	9131
anti-CD4 (EP204), rabbit monoclonal	Cell Marque	104R-24
anti-CD8 (SP16), rabbit monoclonal	Spring Bioscience	M3162
anti-CD19 (SP16), mouse monoclonal	Bio-Rad	MCA2454
anti-STAT3 (124H6), mouse monoclonal	Cell Signalling	9139
anti-pY705-STAT3 (D3A7), rabbit monoclonal (for IF)	Cell Signalling	9145
anti-Cytokeratin, pan (AE-1/AE-3) [Alexa Fluor® 647], mouse monoclonal	Novus	NBP2-33200AF647
anti-Bcl-xL, rabbit polyclonal	Cell Signalling	2762
anti-Mcl-1 (D35A5), rabbit monoclonal	Cell Signalling	5453
anti-GAPDH (6C5), mouse monoclonal	SantaCruz	sc-32233
anti-VEGF (C-1), mouse monoclonal	SantaCruz	sc-7269
anti-α-SMA (Smooth Muscle Actin), rabbit polyclonal	Abcam	ab5694
anti-cyclin D1 (A12), mouse monoclonal	SantaCruz	sc-8396
anti-α-Tubulin (GT114), mouse monoclonal	GeneTex	GTX628802
Secondary antibodies		
anti-mouse IgG, HRP-linked	Cytiva	NXA931V
anti-rabbit IgG, HRP-linked	Cell Signalling	7074
anti-mouse IgG, [Alexa Fluor® 555]	Invitrogen	A-31570
anti-rabbit IgG, [Alexa Fluor® 555]	Invitrogen	A-31572
RT-PCR primers		
Human GAPDH	RealTimePrimers.com	VHPS-3541
Human BIRC5	RealTimePrimers.com	VHPS-830
Human MMP9	RealTimePrimers.com	VHPS-5769
Human CCND1 F: 5-GAAGATCGTCGCCACCTG-3	This paper	N/A
Human CCND1 R: 5-GACCTCCTCCTCGCACTTCT-3	This paper	N/A
Human BCL2L1	RealTimePrimers.com	VHPS-788
Human VEGFA	RealTimePrimers.com	VHPS-9899
Human IFNG	RealTimePrimers.com	VHPS-4477
Human GZMB	RealTimePrimers.com	VHPS-3944
Human IL10	RealTimePrimers.com	VHPS-4508
Inhibitors		
Olaparib	Selleck Chemicals	S1060
Napabucasin	Selleck Chemicals	S7977
